# Small Cell Variant of Medullary Thyroid Carcinoma: A Possible Treatment

**DOI:** 10.7759/cureus.9305

**Published:** 2020-07-20

**Authors:** John Sherret, Mohammad Alomari, Joshua Coleman, Agnes Hamati

**Affiliations:** 1 Internal Medicine, East Tennessee State University Quillen College of Medicine, Johnson City, USA; 2 Oncology, James H. Quillen Veterans Affairs Medical Center, Johnson City, USA

**Keywords:** small cell variant, medullary thyroid carcinoma, levothyroxine, octreotide, nausea, vomiting, hypothyroidism, multiple endocrine neoplasia, calcitonin, thyroid-stimulating hormone

## Abstract

Small cell variant of medullary thyroid carcinoma is an extremely rare histologic entity with a paucity of data. As such, there is a lack of literature and clinical experience regarding this disease. In this report, we examine a case of small cell variant of medullary thyroid carcinoma that presented with intractable nausea, vomiting and diarrhea. While these symptoms were essentially refractory to the standard symptomatic treatment, further laboratory analysis revealed dramatically elevated calcitonin levels and mildly raised thyroid-stimulating hormone levels. Interestingly, repletion of thyroid hormone and treatment with lanreotide resulted in an abatement of our patient’s symptoms. This temporal clinical improvement highly suggests a potential role involving thyroid-stimulating hormone and calcitonin levels in the pathogenesis of this disease, and consequently suggests a role for thyroxine in treating the associated gastrointestinal symptoms.

## Introduction

Medullary thyroid cancer is a neuroendocrine tumor that originates from the parafollicular C cells of the thyroid. It can occur sporadically as in 75% of cases or it can occur in multiple endocrine neoplasia syndromes [1]. It comprises only 5%-10% of thyroid malignancies [2]. Among these malignancies, small cell medullary thyroid cancer (SCMTC) is a rare variant with a poor prognosis [3]. SCMTC is typically an aggressive form of the cancer that requires aggressive treatment. Unfortunately, there is a lack of literature and clinical experience in dealing with this variant. The national library of medicine contains some research usually pertaining to pathology. The prognosis of SCMTC in a large cohort has not been well reported in the literature [4]. In this case, we present a possible mechanism of controlling nausea, vomiting and diarrhea in this patient population. In addition, we aim to report a possible direct relationship between thyroid-stimulating hormone (TSH) and calcitonin levels in this disease. 

## Case presentation

A 52-year-old male presented to the emergency department with diffuse, nonradiating abdominal pain of three-day duration. In addition, there were about four episodes of nonbloody and nonbilious emesis daily. The patient also reported 10-15 bowel movements consisting of clear water daily. The patient denied any alleviating or exacerbating factors. 

His past medical history was significant for medullary thyroid cancer secondary to multiple endocrine neoplasia type 2A diagnosed in 1994. His disease was complicated by recurrence in 1998, 2005 and finally in 2018 for which he received palliative chemotherapy with carboplatinum/VP-16, which showed improvement in his left cervical lymphadenopathy. His last positron emission tomography (PET) scan in 2019 revealed clinical stable disease with a favorable prognosis. Notably in 2012, he was complaining of chronic diarrhea which was successfully controlled with prescribed octreotide and promethazine. In 2018, the biopsy of the recurrent cancer revealed small cell variant medullary thyroid carcinoma (MTC).

He had no known allergies and did not smoke or drink alcohol. His home medications included codeine 30 mg for diarrhea, hydrocodone 5 mg/acetaminophen 325 mg for pain, promethazine 25 mg for nausea and vomiting, synthroid 0.112 mg and lanreotide 120 mg subcutaneous once every four weeks with the most recent dose due just prior to admission. Lanreotide was used in place of octreotide due to policy changes at the Veterans Affairs. 

On examination, his weight was 63.14 kg and his height was 170.2 cm, giving him a body mass index of 21.8 kg/m^2^. Blood pressure was 131/91 mmHg, pulse was 116 bpm, oxygen saturation was 99% on room air, respiratory rate was 20 breaths per minute and temperature was afebrile at 36.6 degrees Celsius. He was alert and oriented but was in discomfort and exhibited multiple episodes of retching in the examination encounter. His abdomen was soft but diffusely tender. However, there was no rebound tenderness or rigidity. 

Laboratory investigations revealed a mildly elevated white blood count of 14,000 cells/mcL. The hemoglobin was 11.6 g/dL on admission; however, it gradually declined to plateau around 8 g/dL after an admission period of four weeks with daily blood draws. Comprehensive metabolic panel was unremarkable with a normal lipase level 20 U/L. He had chronic gastrointestinal blood loss with a mean corpuscular volume of 76.2 fL. Prior colonoscopy a year ago had only shown a rectal ulcer. 

**Figure 1:**
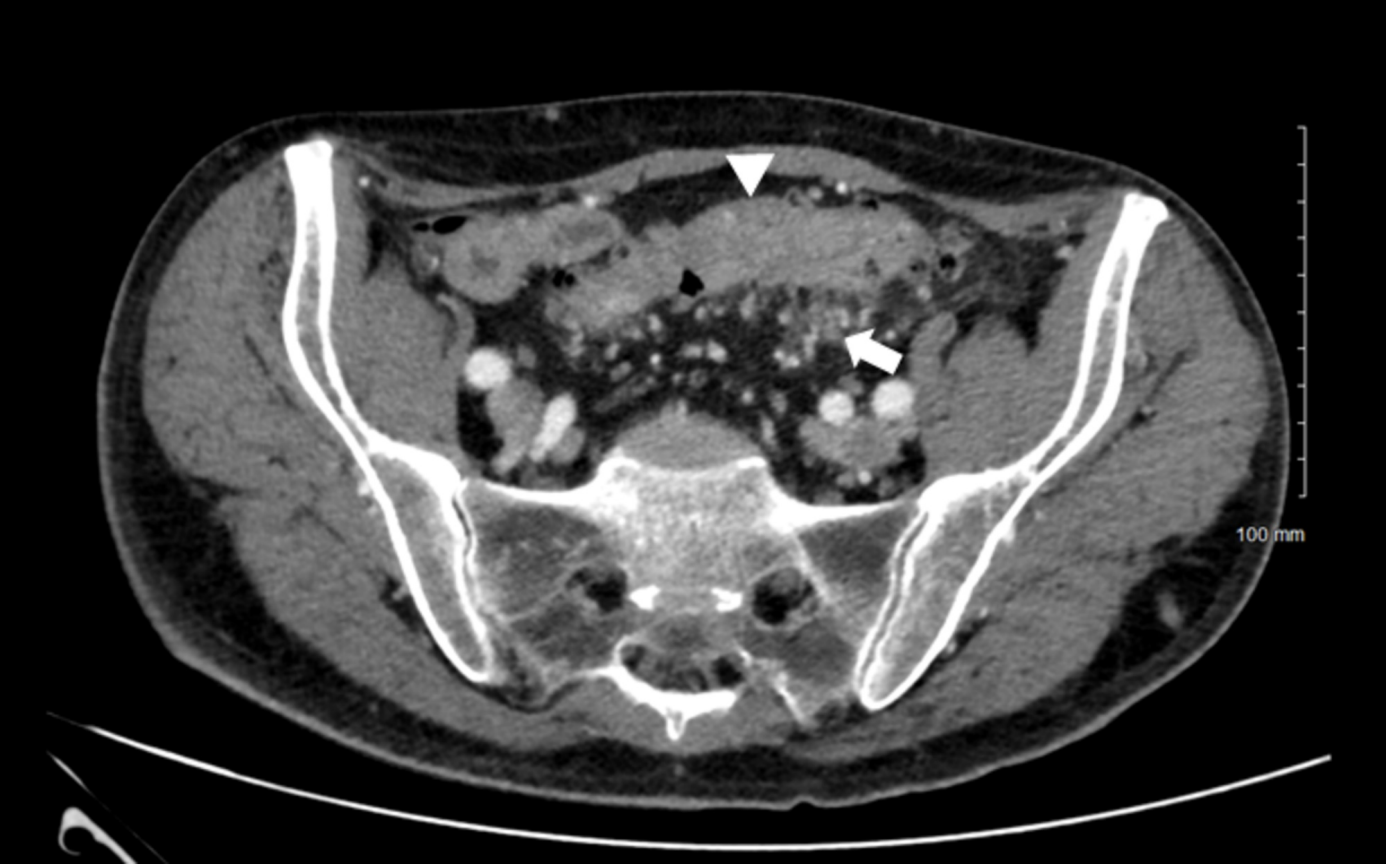
CT of the abdomen obtained two days prior to admission shows wall thickening and perisigmoid fat stranding. Wall thickening is marked with an arrow head and the perisigmoid fat stranding is marked with an arrow.

Imaging of the abdomen suggested colitis and revealed changes in liver nodules that had been detected six months earlier (Figure 1). Some of these nodules were larger, while some had gotten smaller. The liver function tests were unremarkable. He was admitted for intractable nausea and vomiting. He was given synthroid 0.224 mg daily. Phenergan, zofran and a clear liquid diet were given. He continued to have symptoms. Gastroenterology, surgery and psychiatry observed the patient, and all recommended conservative therapy. When there was no improvement within a week gastroenterology performed an esophagogastroduodenoscopy, which revealed antral erosions, gastric inlet pouch and an irregular Z-line. The patient would improve enough to tolerate clear liquids only to have the progress lost by returning abdominal pain, nausea and vomiting. This cycle occurred for approximately three weeks.

**Figure 2:**
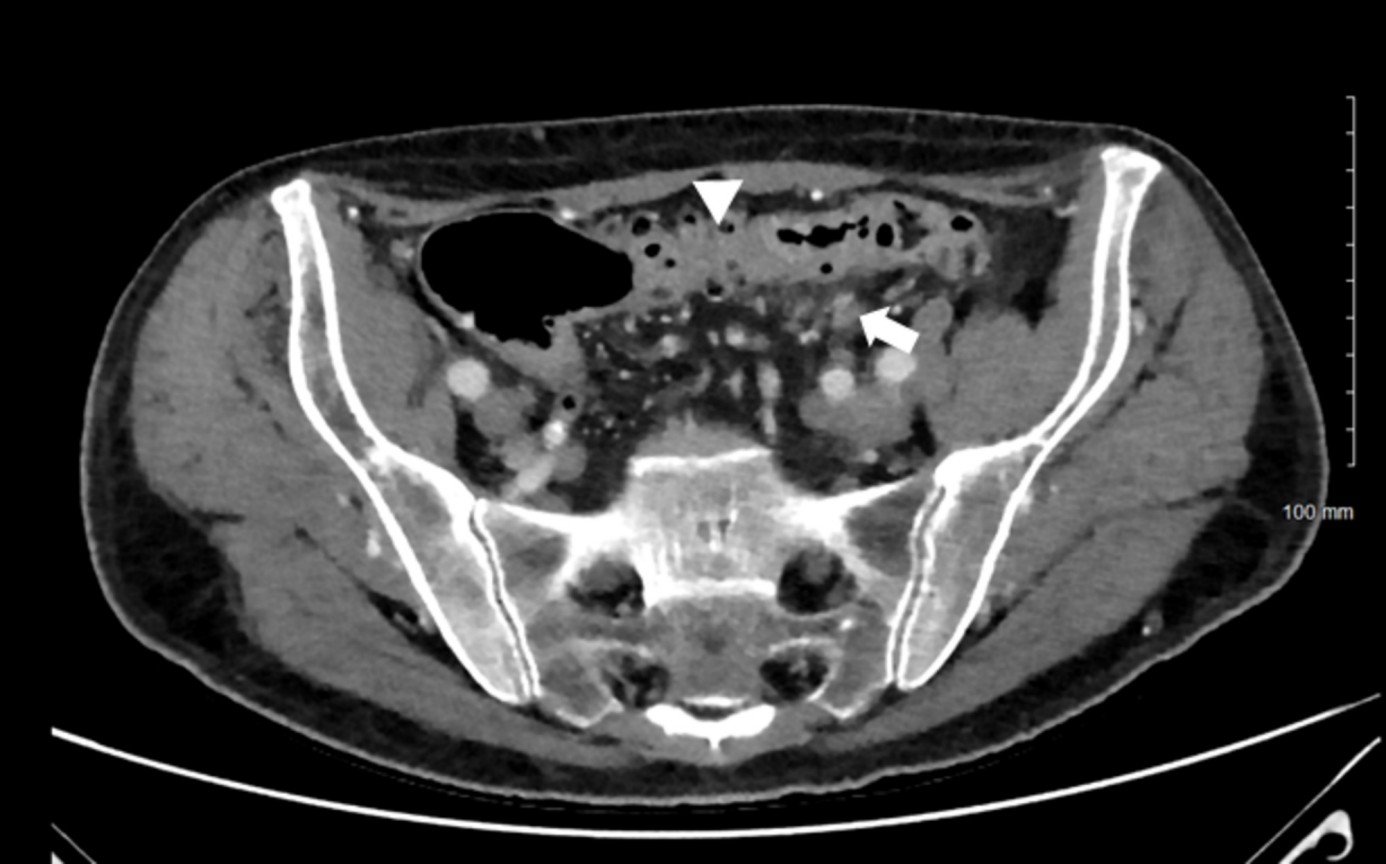
CT of the abdomen obtained after alleviation of symptoms continues to show perisigmoid fat stranding and wall thickening. Wall thickening is marked with an arrow head and the perisigmoid fat stranding is marked with an arrow.

The abdominal pain was finally resolved after a subcutaneous nerve block was attempted; however, the nausea, vomiting and diarrhea persisted. Oncology was consulted and did not believe that the patient’s symptoms were due to progression of MTC. Various medications such as scopolamine, metoclopramide, marinol, dexamethasone and withholding of pain medications were tried with variable results. A nasogastric tube was attempted and then a transpyloric tube feed was started with fluctuating results. Oncology was consulted again and recommended to switch lantreotide to octreotide and to give supratherapeutic intramuscular levothyroxine to depress the TSH. After implementation of this plan, the nausea and vomiting rapidly improved. The patient was finally able to tolerate a consistent carbohydrate diet. His symptoms did not relapse after the implementation of this plan. Repeat abdominal imaging was unchanged from initial scans (Figure 2). 

After discharge, a specific set of labs were ordered prior to the patient’s outpatient oncology appointment. A complete metabolic panel, TSH and serum calcitonin were redrawn. During his hospital stay, the TSH was 6.658 uIU/mL and the calcitonin was 64,059 pg/mL. He was discharged with synthroid 0.112 mg tablets oral twice daily, octreotide depot 30 mg every three weeks and a dexamethasone taper. Repeat measurements two weeks after discharge showed TSH equal to 1.058 uIU/mL and calcitonin now down to 37,213 pg/mL (normal 0.0-8.4 pg/mL). 

Two weeks after discharge, the patient reported continued cessation of symptoms. He was on schedule with his medications. His abdomen was soft and nontender. 

## Discussion

MTC is a malignancy of the parafollicular cells of the thyroid gland. MTC comprises approximately 5% of all thyroid cancers. The majority of MTC cases present as a solitary thyroid nodule [5-8]. The 10-year survival for MTC is 71%-87% [9]. SCMTC however is a histological subcategory of MTC with the first case being diagnosed in the 1980s with advances in immunohistochemistry. SCMTC is typically aggressive and denotes a poor prognosis [3]. It is more lethal than MTC and has a mortality rate similar to anaplastic thyroid cancer [4]. One pathology case report noted a patient who was diagnosed with SCMTC and treated with radiotherapy only to find metastasis three months after treatment. Various other reports on SCMTC recommend aggressive surgical or radiation therapy [3,4,9,10]. 

Elevated calcitonin levels have been shown to cause diarrhea. The most common presentation of MTC is a palpable thyroid nodule [5-8,10]. Patients can also present with diarrhea as the initial symptom, which is a consequence of calcitonin excess [10,11]. Patients with chronic secretory diarrhea should be screened for endocrine active tumors [12]. Following diagnosis of MTC up to 30% of patients will develop diarrhea as this usually means metastasis and excess calcitonin as a result [13-15]. In one case of MTC, a patient presented with diarrhea and a calcitonin level of 19,315 ng/L (normal 0-11.1 ng/L). The patient received total thyroidectomy, right selective neck dissection and thymectomy with a resulting reduction of serum calcitonin to 885 ng/L and complete resolution of the diarrhea [16]. This demonstrates the utility of surgical resection in treating the diarrhea and also shows that extreme elevations of calcitonin are required for diarrhea to manifest. In another case, a 52-year-old male presented with diarrhea of 15-month duration and a calcitonin level 4,572 ng/L. After resection of the tumor, the calcitonin dropped to near normal levels and the diarrhea resolved [12]. 

SCMTC also appears to demonstrate a relationship between diarrhea and calcitonin. Prior cases involving SCMTC without diarrhea have noted normal or mildly elevated calcitonin levels [3,17]. In the present case, our patient was found to have diarrhea with a calcitonin level of 64,059 ng/L. Repeat laboratory evaluation after the resolution of watery diarrhea showed that the calcitonin level had dropped to 37,213 ng/L. This suggests that high relative calcitonin levels in SCMTC are predictive of diarrhea just as they are in other forms of cancer. Octreotide and other somatostatin analogs such as lanreotide have been recommended to help treat the symptoms of refractory diarrhea in MTC cases. These analogs have not been shown to decrease or change the calcitonin levels [18,19].

Interestingly, a hypothyroid state can rarely present with nausea, vomiting, diarrhea and abdominal pain in contrast to the usual clinical presentation of constipation. Sweet et al. presented a case of a 45-year-old female with intractable nausea, vomiting and diarrhea but no constipation [20]. Thorough investigations were unrevealing until an elevated TSH was observed. Thyroid hormone was given and the symptoms resolved. In our case, the patient was experiencing similar symptoms and was found to be in a hypothyroid state. The symptoms likewise disappeared after he received thyroid hormone replacement therapy and octreotide.

In our patient, colitis was considered in the differential but was ultimately dismissed as the cause of symptoms. Although our patient presented with clinical and radiological signs of colitis, he underwent three CT studies of the abdomen that did not demonstrate radiographic improvement following clinical resolution. The first image showed perisigmoid fat stranding and wall thickening (Figure 1). A second image obtained during the middle of the hospital course showed mild improvement but also progression in some areas. A third and final image obtained just prior to discharge showed no interval changes (Figure 2). Despite the lack of significant changes between these images, the patient’s symptoms resolved after the reinstitution of thyroxine and octreotide. Given these observations, the diagnosis of colitis was dismissed as the cause of the symptoms. 

Thyroxine suppression therapy is a possible novel treatment for SCMTC. Such therapy is thought to be effective in suppressing differentiated thyroid cancer growth and recurrence by depressing TSH levels. Thyroxine suppression therapy has previously been discouraged in MTC due to the lack of TSH receptors in the parafollicular cells. However, such recommendations have not been described in SCMTC. Various studies involving pathology have been conducted on SCMTC, but very few clinical studies have been conducted presumably due to the rarity of this variant tumor. Therefore, the effect of thyroxine hormone suppression in SCMTC has yet to be determined. Our case suggests a directly proportional relationship between TSH and calcitonin levels which is supported by the remarkable decrease in both calcitonin and TSH levels after administration of thyroxine therapy. The resolution of symptoms further suggests that the high calcitonin levels were mainly responsible for the gastrointestinal manifestations taking into account the temporal improvement after the calcitonin levels dropped. As previously stated, octreotide was also administered but was unlikely to have had any effect on calcitonin levels as outlined in prior studies. 

## Conclusions

This patient with SCMTC, who presented with intractable nausea, vomiting and diarrhea, was refractory to the standard symptomatic treatment. It was finally discovered that the patient had dramatically elevated calcitonin levels and mildly elevated TSH levels which upon repletion of thyroid hormone and treatment with lanreotide resulted in complete abatement of our patient’s symptoms. This finding suggests a pivotal role involving TSH and calcitonin in the pathogenesis of this disease, and consequently suggests a role for thyroxine in treating the associated gastrointestinal symptoms.
